# Growing up HIV-positive in Uganda: “psychological immunodeficiency”? A qualitative study

**DOI:** 10.1186/s40359-017-0199-7

**Published:** 2017-08-25

**Authors:** Birthe Loa Knizek, James Mugisha, Joseph Osafo, Eugene Kinyanda

**Affiliations:** 1 Norwegian University of Science and Technology, Faculty of Health and Social Science, NO-7491 Trondheim, Norway; 2 James Mugisha, Kyambogo University, Kampala, Uganda; 3 University of Ghana, Legon, Accra Ghana; 4 MRC/UVRI Uganda Research Unit on AIDS, Entebbe, Uganda

**Keywords:** Adolescence, HIV, Uganda, Mental health

## Abstract

**Background:**

This study is part of a longitudinal study among children and adolescents with HIV in both urban and rural Uganda: *‘Mental health among HIV infected CHildren and Adolescents in KAmpala and Masaka, Uganda (CHAKA)*’.

**Method:**

The study is constructed of both quantitative and qualitative components. In this article we report a qualitative study on the experiences of 21 adolescents (twelve to seventeen years) living with HIV in Uganda. The purpose of the study was to investigate both the protective and the risk factors in HIV-infected adolescents’ care environment in order to understand what might contribute to negative outcomes and what might provide a protective buffer against harmful life events. Semi-structured interviews with vignettes about mental disorders were employed and a phenomenological analysis was done.

**Results:**

The findings uncovered that the adolescents’ families were mostly characterized by instability and diffuse relationships that provided an insecure basis for secure attachment and emotional support. Even in stable and secure family environments, there was no guarantee for getting sufficient emotional support in order to develop a positive self-concept due to the fate being the only infected child in the family. Both secure attachment and positive self-concept are known psychological protective mechanisms that provide the individual with resilience. The adolescents in this study seemed hampered in the development of protective mechanisms and consequently seemed psychologically vulnerable and badly equipped for coping with challenges, which paves the way for the possible development of mental disorders.

**Conclusion:**

To change the focus towards strengthening the children and adolescents’ development of psychological protective mechanisms implicates a change in focus from illness to health and has consequences for both treatment and prevention. Psychological health promotion must be systemic and aim at strengthening the family environment, but also to establish peer group support.

## Background

The Joint United Nations Programme on AIDS (UNAIDS) estimates that 90% of the over 2 million children living with HIV globally reside in sub-Saharan Africa [1]. In Uganda, the country with the fifth highest prevalence rate in the region, 150,000 of the 1.2 million people living with HIV are under 15 years of age [1]. Many of these children and adolescents are both infected and affected, as they have the double burden by being single or double orphaned in addition to living with a chronic illness. It has been found that HIV in children and adolescents is associated with psychiatric disorder in addition to the physical impact of HIV and possibly delayed/impaired physical and cognitive development [2]. Epidemiological studies from high-income countries have established that HIV-infected children and adolescents experience high rates of psychiatric disorders with estimates ranging between 27 and 61%. The most commonly reported psychiatric disorders among this group are behavioural disorders (e.g. attention deficit hyperactivity disorder), and emotional disorders (e.g. anxiety and depressive disorders) [3–8] Psychiatric disorders among children and adolescents with HIV leads to psychological distress and impaired quality of life, increased stigma and isolation and may lead to negative clinical and behavioral outcomes. Despite antiretroviral therapy improving the survival of HIV-infected children, the mental health problems of these children and adolescents have had less attention [9].

Although sub-Saharan Africa shoulders the greatest burden of HIV, few studies have investigated psychiatric problems among children and adolescents with HIV in the region. In 2009, Musisi & Kinyanda observed that 51.2% of CA-HIV in Kampala, Uganda exhibited above threshold psychological distress scores [10]. The most common diagnoses were depression, anxiety and somatisation. In a more recent Kenyan study, Kamau and colleagues [11] reported a rate of 48.8% for at least one psychiatric disorder with the most reported psychiatric disorders being depression, social phobia, oppositional defiant disorder and attention deficit hyperactivity disorder.

In order to come to a closer understanding of these children and adolescents we need to study their interplay with their environmental conditions as possible outset for the development of psychological vulnerability. In the Ugandan context the extended family is the fundamental social security net for children and adolescents, who are in transient relationships [12–15]. Often these families are impoverished and have a high dependence burden [15, 16]. Despite the informal social security net being of indisputable value for the survival of these children and adolescents, it is necessary to look at the fundament it creates for the children and adolescents’ cognitive and emotional development, as this influences how they will be able to tackle both living with a chronic, stigmatizing illness, new life events and forming a future in a society, where they are expected to get a family.

In addition, we have to consider that these children often are both ill and orphaned (infected and affected), which also influences the developmental environment. Moreover, Abebe & Skovdal [17] have introduced the concept of social orphans describing children vulnerable to illness and parental illness, which applies to the adolescents in this study. Their care trajectory often is turbulent and unpredictable, which might affect their attachment history, identity formation and robustness against additional life events that might be multiple. In addition to their own considerable health problems they struggle with “…the AIDS stigma, the prolonged terminal illness of one or both parents, siblings and other relatives, bereavement and meeting their own survival needs as orphans. As would be expected, this has a detrimental impact on children’s psychosocial health and well-being at the level of the household and community and places children and young people from AIDS affected households at risk of trajectories of social exclusion” [18]. With this multitude of possible challenges, it is important to investigate both the protective and the risk factors in their care environment in order to understand what might contribute to negative outcomes and what might provide a protective buffer against harmful life events.

Kamali et al. [19] found that orphans generally seemed taken good care of by the community, which is in line with other studies [13, 20] that find the orphans satisfactorily looked after as their basic needs are met by their extended family, even though it sometimes is in extreme deprivation [21]. Oleke et al. [21] have pointed out that the view on how orphans are catered for during the AIDS epidemic might be somewhat optimistic and Baylies [22] describes the extended family as “safety net with holes”, as the protection and care offered depends on an individual’s status in the network. In addition, Oleke et al. [23] have pointed out that in most of sub-Saharan Africa property is inherited through the male line, which makes children that grow up with their maternal kin unsure about where they belong and which rights they have especially regarding their property.

Moreover, Ansell & Young [24] have in their studies in Lesotho and Malawi shown that most orphans experience multiple migrations and that they in the beginning feel this traumatic, but in the long term settle in. Given all these challenges both in their past, actual situation and future, it is important to study the adolescents’ own experiences of their situation, especially as approximately half of the HIV-infected adolescents display symptoms that are consistent with behavioral and mood disorders. This study is part of a longitudinal study among children and adolescents with HIV in both urban and rural Uganda: *‘Mental health among HIV infected CHildren and Adolescents in KAmpala and Masaka, Uganda (CHAKA)*’ (the acronym “*CHAKA*” means, “to search” in the local Bantu dialectic).

## Methods

### Study setting

This research project is a sub-study of the mental health problems of children and adolescent with HIV study in Uganda (CHAKA). The qualitative sub-study was undertaken at 4 children and adolescent HIV clinics and one psychiatric hospital. Two of the HIV clinics were placed in the urban centre of Kampala (JCRC and Nsambya) and two at semi-urban/rural sites in Masaka (TASO and Kitovu Mobile). The psychiatric hospital (Butabika) is the only of its kind in Uganda and placed in Kampala. Kampala is the capital of Uganda and has an estimated population of approx. 1, 7 million people, while Masaka district has an estimated population of 300.000 [25]. The main source of income in Masaka district is agriculture and farming. The normative context in Uganda is a strong patriarchy [26] and very religious [27] with dominantly Christianity (85.2%) and Islam (12.1%) [28].

### Study participants

Inclusion criteria were adolescents between 12 and 17 years, being tested HIV positive. Respondents included 21 adolescents (nine boys and twelve girls) with HIV with 10 adolescents from rural and urban sites respectively, as well as one participant admitted to a psychiatric hospital. Details described in Table 1.

**Table 1:** The participants and their family context

Clinic	Gender	Age	Orphan (double/single)	Stay with 2 parents	Stay with 1 parent	Stay with grandparent(s)	Stay with others
Urban 1	4 girls, 1 boy	14-17	4	1	0	1 (Maternal)	3
Urban 2	3 girls, 2 boys	12-15	3	1	1 (Father)	2 (Maternal/paternal	1
Rural 1	2 girls, 3 boys	13-16	3	0	5 (Mother)	0	0
Rural 2	2 girls, 3 boys	13-17	5		1 (Mother)	2 (Maternal)	2
Mental hospital	1 girl	16	1	0	1 (Mother)	0	0

The mean age was 14.6, but some of the respondents did not know how old they were. Fourteen of these adolescents were either single or double orphans. Two other adolescents did not know whether their father was alive, since there was no contact. Of the four adolescents (2 boys and 2 girls), where both parents were alive, only two lived with both their parents (one boy and one girl). Of the single orphans, eight lived with their mother and/or others, while only three single orphaned lived alone with their parent. Sixteen of the adolescents lived in a household with their maternal kin, while one lived with total strangers. The adolescents lived in households with 2 to 10 members. Six of the informants reported that they had been starving sometimes during the past 30 days. All but three went to school and were in classes from primary three to senior three, but the classes visited were not necessarily according to age. One girl and two boys had left school after primary seven and were now unemployed. One informant had been admitted to a psychiatric hospital.

### Study design

We conducted 21 interviews with adolescents on basis of a semi-structured interview guide. The interview questions touched on background, household composition, economy, family activities, responsibilities and friends. In addition, every adolescent was presented a vignette of an HIV positive child or adolescent with mental problems (ADHD, depression, cognitive impairment, conduct disorder) and was asked about which problems he/she saw in the story, what kind of help would be needed, who could provide the necessary help, how he/she assumed the reactions of people towards this child/adolescent and which consequences the problems could bear in the long run. These vignettes were used in order to help the adolescent to reflect on own situation and help available.

### Study procedures

Adolescent recruitment in the study was random for those coming to the HIV clinics for treatment and care. After going through the clinic procedures, the clinic nurse directed them to the researcher for information and recruitment into the study. Few adolescents came with caregivers to the clinic. Those coming with the caregivers were given information together with the adolescent and asked to participate in the interviews and consent was obtained in written form. Adolescents who came without caregivers to the clinic were given the information sheet to take back to the caregivers. Those willing to participate came back to the HIV clinic with the adolescent. After signed consent, the interviews were mostly done in the local language, Luganda, apart from three that were done in English. The interviewer was a trained researcher speaking both Luganda and English. All interviews were recorded, transcribed and translated into English by the interviewer [29, 30].

### Data analysis

As the focus of the study was the experienced life world of the adolescents, a phenomenological approach [24] was chosen for the analysis. Prior assumptions (like the indisputable value of the extended family) were bracketed off, while the interview was broken down into relevant meaning units and interpreted in a hermeneutical circle between the parts and the whole interview. The analysis, categorizations and interpretations were done by the first author and discussed with the other authors during the process.

The informants will be presented by means of urban (U) or rural (R), gender (m/f) and age only in order to preserve their anonymity.

### Methodological considerations

The interviews were relatively short and lasted between 31 min and 13 s and 12 min and 5 s. The average was about 19 min. Despite the short length of the interviews, the participants managed to express surprisingly “rich texts” with personal descriptions. Seemingly, the concrete but open questions on how and whom they lived with, their daily struggles and pleasures provided a comfortable basis for telling their own story of their care environment. We adhered in this study to the COREQ guidelines/methodology.

### Ethical considerations

Ethical and scientific clearance for this study was sought and obtained from the Science and Ethical Committee of the Uganda Virus Research Institute, the Uganda National Council of Science and Technology and the Science and Ethical Committee of the London School of Hygiene and Tropical Medicine. Signed consent was obtained from all caregivers of all eligible children and adolescents with HIV. Signed assent was obtained from all participating adolescents (12–18 years) living with HIV/AIDS. HIV positive study respondents were assured that details of any risk behaviors would not be reported to their caregivers. It was made clear to all participants that refusal to participate in this study would not have any negative impact upon their treatment and care. Due to the anticipated psychological distress, which some of the interview questions were likely to create, all research staff with direct involvement with study participants received training on how to sensitively deliver interviews and handle situations in which sensitive information is disclosed or emotional distress observed. Participants found to have a psychiatric disorder were referred to mental health clinics nearest to them. In cases of psychiatric emergencies, e.g. highly suicidal individuals or individuals with severe depression, research staff (who were all mental health workers) provided emergency intervention and referral to psychiatric hospitals.

## Results

By means of the analysis, two key findings seemed significant for the development of psychological resilience factors of the adolescents: “Family form and stability”, which deals both with the volatility of the adolescents’ care environment and their perception of their familiarly relationships both having possible consequences for the development of secure attachment. “Emotional support” that goes into whether the psychological needs of the adolescents are met in the family and the adolescents’ possibility to build up a positive self-concept. In addition, the adolescents report on how they cope with their situation.

### Family form and stability

During the interview, our informants were asked about their family composition, which many had difficulties to answer. While many of the family members spent the day together for work and meals, they would sleep in different houses. As the family seemed to change form during the 24 h the informants not always knew who to count in their family. In addition to the shifting numbers, the labelling of the relationship with their caregivers could be complicated. For example, they could talk about their grandmother, which then turned out to be a remote aunt or not even a blood relative at all.

Int.: “Is grandmother your maternal or paternal relative?”

Resp.: “No, she is sister to my mother.”

Int.: “Is she not your aunt?”

Resp.: “That is what I was told, that we call her grandmother. Even my older brother calls her grandmother.” (Ub13)

The boy has not chosen the title grandmother himself in order to signal the closeness of this relationship, but was told to do so and this name could be either an honorary title or the relatives’ attempt to integrate this double orphan in a close family relationship. On the question whether they ever spend time as family together he answers negatively, “Some go to the village and leave some of us behind”. In this family the boy gets his basic physical needs fulfilled, which was not the case, when the father was alive and the family had to starve. According to Kamali et al. [19] and Foster [13, 20] his actual situation could be described as satisfactorily taken care of, and better as when he was staying with his father. We do not know, however, whether his psychological needs are met or whether the fact that he is staying with maternal kin provides an insecure future as pointed out by Oleke et al. [23] and which psychological consequences this might have.

Another example is the fifteen-year-old girl that elaborated her relationship to her family: “I stay with grandmother and grandfather, my paternal aunt and that one; father” (Ug 15) and the total number of people living together were six. Later, when she spoke about the father, she made an additional precision: “This is not my biological father. My biological father stays far away”. In this case, we do not know who “that one” is or whether and how the paternal aunt and the person, she calls father, are related and in which way she is related to them. These six people were spending their days together, but spent the night in three different houses. The label “father” seems a social title instead of a description of the actual relationship and we do not know anything about the emotional qualities of the relationship. Chirwa [31] who has introduced the concept of senior and junior fathers, meaning brothers of the father that are either younger or older and taking over the responsibility, might provide an explanation and we might expect the girl’s father to be a paternal uncle.

Equally, aunts and uncles were frequently mentioned without necessarily being blood relatives. A 16-year-old girl explained her relationship like this: “I call her aunt because she adopted me” (Ug 16). This girl’s care trajectory went from living with her sick and widowed mother and three siblings until mother died when she was six years-old. She then stayed with an aunt, who exploited her as housemaid and took all the money she earned. The aunt was practicing witchcraft, which made a lot of additional work (ashes and salivary in the house), which the girl was supposed to clean up in addition to her job as housemaid. Her next home was with grandmother, who had to take care of too many children and could not pay their school fees and the girl had to leave school. Later an unrelated single man, who took photos of sponsored children, who she was one of, noticed her. He brought her to an orphanage and paid for her school fees. Because of him taking over the financial responsibilities, she declares that his family raised her. She stayed in the orphanage, where she was stigmatized and isolated until the man was married and settled, whereafter she moved in with his family. At the time she moved in, the wife of the man already had two children. Sometimes she calls her primary caregiver an uncle and sometimes “Daddy”:

Int: “Which Dad?”

Resp: “The one I call uncle; he is more like a father to me (…).”

She does not know anything about the siblings and has no contact. This girl is one example of a turbulent care trajectory, which is common in a context with high morbidity and poverty that results in work related migration. Despite the traumatic care trajectory, the girl shows by means of the reassignment of “uncle” to the more affectionate title of “Dad” that she feels contented and desires closer relationship with this caregiver. The children and adolescents in our study might be passed on from relative to relative or even people unrelated, like in the latter case. This care trajectory might be traumatizing in itself as pointed out by Mattes [32], but fundamentally, it also might have an impact on the attachment between the children and their caregivers, which again then influences the identity formation and psychological robustness. As attachment is a strategy for prediction and protection of oneself against dangers [33] these children and adolescents might be psychologically vulnerable already on the basis of growing up in unpredictable circumstances, where families change form in the short and long run. Among the 21 participants, only four were having both parents alive and only two lived with them. Nevertheless, since almost all of these adolescents were born HIV positive (one was infected through a blood transfusion), the health status of their remaining parents might be quite fragile, as we would expect most of them to be HIV positive as well. The children and adolescents thus have to live with the constant threat of double orphan hood and an unpredictable future in addition to their own chronic condition and sometimes-severe physical problems. Ansell & Young [24] have shown that most orphans despite multiple migrations, which they in the beginning feel traumatic, settle in. Despite this positive finding we have to question whether the volatile family environment in which the children and adolescents live has influenced their development. The result might be a psychological vulnerability where they become prone to develop mental disorders, even though they settle in, due to the failure of developing successfully protective psychological factors.

The constant shifts in care environment, perception of family and unpredictability of the future could result in some insecurity, which we found for example in the case of another 15 years-old girl that lives alone together with her grandmother. Her mother is dead and she answers the following on a question regarding her father: “I last saw him when he told us that he is not our father and now we are hearing that he is in Sudan. Some say that he is dead. Everything is confusing so we just let it go” (Ug 15). She was told that she has many siblings, but only knows two that are older and live somewhere else. She is the only one of the siblings, who is HIV positive. Her anxiousness about her primary caregiver and future became obvious when asked about getting help to problems:

Resp: “(…) But some problems I do not tell her because she does not have money, she will panic yet she is already sick. Some things I keep to myself. You can tell her how tough books are and she tells you they went through it too.”

Int: “Hmm okay. So, what problems don’t you mention to her?”

Resp: “I have never told her that often times my ears block and something covers my eyes and I can’t see. If you tell her such things, she panics and gets high blood pressure attacks.”

Int: “Hmmm”

Resp: “According to what I saw once, I decided to always keep some things to myself.”

This girl, like many of the other informants, struggles with neurological complications of HIV/AIDS and with severe side effects from the ARV treatment as the impoverishment of the families means that they do not get as sufficient healthy food, rest and medical referrals for additional HIV complications. That means that if the girl mentions her physical problems, the grandmother will panic because of the futility of getting better finances, which then again could worsen her health condition. Grandmother’s doctor thus had cautioned the girl not to tell her issues that could make her tense. Grandmother and granddaughter live on some remittance that an aunt gives them on an irregular basis and sometimes they have to starve. The school fee is only paid, when the girl has been chased away from school. This girl lives in an insecure environment, where neither the material nor psychological needs are fulfilled. She keeps silent about her own troubles in order to retain some security for the future. However, she is caught up with her fears for own immediate health problems, grandmother’s health, her survival without regular financial support, her future health and possibility of getting married and getting children, as well as the daily life’s stresses of being bullied and stigmatized from schoolmates and teachers. She feels ashamed of being HIV positive and finds it hard to understand that also children can have this disease. Not surprisingly, she narrates suicidal ideation and report some half-hearted suicide attempts in her past and may thus be able to join with people that genuinely care for her: “According to me, when you keep thinking about your past problems, like for me I never had a Dad’s love. It was so difficult so I thought I could die too and meet them there.”

Her wish to die and symptoms of depression that she expresses seem to be a reaction to her insecure living situation and future. According to Sund and Wichstrøm [34] insecure attachment to parents may contribute to the development of severe depressive symptoms among young adolescents. This girl has problems developing any kind of secure attachment, which makes her very vulnerable. However, in her suicidal ideation she expresses death as medium to rejoin her other family members, which indicates a belief in afterlife. From a clinical psychological perspective, it is interesting whether to assess her suicidal expression as a depressive symptom or cultural problem solving strategy. This girl finds the meaning of her condition in her religion and by normalizing and universalizing the situation: “It must have been God’s plan that I would be HIV positive, but I am ok with it; after all, everyone has it anyway.” She experiences the situation not as manageable, but probably as comprehensible by referring to her religious belief and narrates suicidal ideation as a way of rejoining with her close family and paternal kin. This girl thus has developed a coping strategy that, unfortunately, might lead to suicide.

Common for the adolescents described above is that they grow up in shifting family conditions, as their parents are dead. This results in insecurity and influences the possibility for a healthy psychological development, as it is questionable whether many of them ever will be able to develop sufficient attachment to their shifting caregivers. Likewise, Oleke et al. [21] have studied the care environment for orphans in Northern Uganda and shown how different contexts (maternal/paternal kin for example) provide different life and thus developmental conditions. Many children and adolescents thus grow up in transient relationships, which might change too often, making it impossible for them to develop secure attachment. The “safety net with holes” [22] might seem to be an insecure basis for the development of sufficient psychological resilience necessary to tackle the many problems that these young people encounter, thus paving the way for the development of mental disorders. However, we also need to look at the quality of the relationships or emotional support that the adolescents get from their caregivers.

### Emotional support in the family

Many of our informants were the only in the family born with HIV, which would give them a special status in the family, independent of whether they were living with close relatives or not. As earlier pointed out it depends on the status of the care receiver, what kind of help s/he will get from the social network [22]. Our interviews revealed that complicated family dynamics in addition might be at play. An example of this is the girl (Ug 17), who is one of the two informants, who lives with both parents and thus should have a secure basis compared to others with a turbulent care trajectory. Her status as only HIV positive child among her siblings was difficult to accept for the mother:

“(…) I grew up when my mother never saw me as a person who can really achieve something in future, because I am the only kid who was born HIV positive. (…) So, she saw me like a failure, I would not succeed in anything. (…) She used to discriminate me among my brothers and sisters. She used to treat them as children, but me as nothing. A bastard at home.”

There might be different reasons for the mother to treat the girl so she feels like a bastard. One reason might be that the mother feels guilty of having transferred the disease to the girl; another might be that she functions as the scapegoat for mother’s anger against father, who might have transferred the disease to the mother and as a result to the girl. The girl struggles with the absence of the maternal love, which seems abnormal:

“(…) I got to know that mothers are the most creatures that really love their children compared to their dads. (…) But I was really surprised that it’s my dad who loves me more than my mum. So I would ask myself why my mother was doing such. At times I would tell myself that this world is nothing for me.”

She does not understand why her mother seems to act abnormal and she develops suicidal thoughts. The mother does not treat her as the other children, does not want to invest in her education and only has negative comments and no support for her. The girl is happy about the paternal support, but she begs for love from her mother. Not only does the mother discriminate and withhold maternal love from her, but she feels also that the mother deprives her a future. This girl, however, had the constant emotional support of her father in her development, which seemed sufficient for developing sufficient confidence and strength to cope with the situation. She called her parents for a meeting, where she asked the mother, whether she was her real mother, since she never had shown her any love. She was wondering whether she was adopted and the mother was forced to raise her, while her real mother was dead:

“I will know that I am staying with parents who not are my real parents; it will be better. But if you are my real parent, it’s high time for you to change, I don’t know really. So my mum was really scared, she felt nervous and suddenly tears flowed out from her eyes.”

The girl had, despite growing up without emotional support from the mother, managed to compensate this by means of the close relationship with her father. In her example, the necessity of one close and stable relationship during growing up is apparent, as she turned out self-confident and strong enough to choose an offensive coping strategy. Positive self-concept has been shown to be a protective factor for example by Masten & Coatsworth [35] and Williams, Anderson, McGee, & Silva [36]. Among our informants, the girl above is the only one showing this kind of psychological strength. Others were struggling with similar problems, where their relatives or parents did not want to invest in their education and thus future, but had not the self-confidence or strength to fight the situation. For example, the boy (Rb 17), who is in a situation, where his mother refuses both contact and to pay his school fees. He too is the only child with HIV in the family and ashamed about that. He is stigmatized and bullied by the villagers, unemployed and sits at home with “shattered dreams”, even though he tells that he has overcome the disappointment. When confronted with a vignette on depression he has no idea of how to help in that situation other than give medication. In contrast to the girl above, he gives the impression of being depressed and apathetic, without hope for the future and without coping skills. The situation is neither comprehensible nor manageable for him and he does not seem to have any motivation or strength in changing the situation.

These two adolescents are exemplifications of lack of support in close and even stable relationships, but with different outcomes. The reasons for the lack of support might be many, indicating complex and conflictual family dynamics. Only one of our 21 informants had an offensive approach, while others would develop a depression, sometimes with suicidal ideation, if they could not find any meaning in their situation:

“Challenge number one, I can say, is being isolated. You start isolating yourself from other people. You feel you do not deserve to be in public and also having stigma. You start having self-stigma. Another thing is losing hope and giving up so quickly and you say, “Ah, I’m something else and I feel I cannot do anymore. I feel I cannot control it anymore.” With this challenge, you feel like you are confused and saying to yourself that “why cannot I just do something and end my life?” I think the challenge is about having self-stigma.” (Ub 14)

This boy clearly blamed the introjection of stigma and hopelessness as cause for his suicidal ideation. He listed up several possibilities of killing himself, but ended up giving advice how to overcome suicidal thoughts and self-stigma:

“The best thing I would say is accepting yourself the way you are. Saying that I can get a second chance; I can say that number one is God, getting committed to God, asking from him each and everything that you want to change in your life. Number two is about seeking help from other people and this may be a close friend, someone you can talk to and explain to him or her about what you are going through (…).”

He points out several strategies to overcome depression and suicidal ideation: self-acceptance, faith, help seeking and disclosure. He is the only informant mentioning several strategies, while others mention just one, if any. The most often mentioned strategies were trusting in God and to normalize or universalize the situation, by confiding the status to a friend and thus get to know of others, who have the same condition. By the last coping strategy, the adolescents would gain a sense of “normality”, since others might be in the same situation as themselves. Interestingly enough, none of our informants blamed their parents for transferring HIV to them, but the fact that the condition was passed on to them from their mother might raise their expectation of support, which then was disappointed.

In summary we find that family form and stability, as well as lack of emotional support hamper or even prevent the development of both secure attachment and positive self-confidence both known as psychological resilience factors. Some of the adolescents manage, however, to describe possible coping strategies, where faith and normalization among peers had the priority.

## Discussion

The purpose of the study was to investigate both the protective and the risk factors in HIV-infected adolescents’ care environment in order to understand what might contribute to negative outcomes and what might provide a protective buffer against harmful life events. In accordance with the focus of the study on the care environment, the interviews mainly focused on the adolescents’ experience of being part of a family and community context. This approach has the advantage on getting valuable information on contextual aspects that seem important for their psychological development.

The informants in our study had to struggle with the double burden of being HIV positive and orphaned, with stigma connected to both conditions. Their care trajectory often was traumatic and turbulent and the families they lived in were constantly changing, thus providing an insecure basis for secure attachment and development. Even if the care environment was stable, complicated dynamics in these AIDS affected families could obscure good psychological development. The lack of stability in care environment and shortcomings in the emotional support to these adolescents are detrimental for their well-being and seem a risk factor for the development of mental disorders as they could not develop sufficient psychological resilience. Confronted with the findings of Musisi & Kinyanda [10] and Kamau et al. [11], who find approximately half of the children and adolescents with HIV displaying psychological distress above threshold, it seems worthwhile to focus on the care environment as possible arena for the development of a vulnerability for mental disorder. According to Kemph & Voeller [37] it is unlikely that the diagnosis of Reactive Attachment Disorder (RAD) “…can be made in the absence of comorbid diagnoses in adolescence because these children usually have symptoms which meet the criteria for other diagnoses, such as attention deficit disorder (ADHD), post-traumatic stress disorder (PTSD), oppositional defiant disorder (ODD), mood disorder, or conduct disorder (CD) by the time they become early or mid-adolescent ages. During development, additional diagnostic criteria for other DSM-IV diagnoses may be observed. Although a comorbid diagnosis of ADHD, ODD, and/or CD might appear to take precedence over RAD, with the burgeoning information in genetics it may be useful to know that RAD was present or may still be present in the symptom complex of an individual patient.” We thus need to change focus and see whether these adolescents actually display symptoms of a Reactive Attachment Disorder (RAD) as this will have therapeutic and preventive consequences.

However, as LeVine and colleagues [38] have pointed out most of the child developmental research has been driven in Western societies by for example psychiatry in seeking the determinants of mental disorder, which might have resulted in a faulty impression of what children in general need. The cultural norms thus must be taken into account, when interpreting specific settings, as it is context dependent on what is seen as natural, normal and necessary [38] in child raising. Since we know that the extended family system is a pillar in the Ugandan society, we might expect different attachment patterns develop than in the West. However, what is expected to be fundamental for the child and adolescent to develop into a mentally healthy adult seems to be stability, predictability and support: “…formation of key relational and attachment capacities after the first 5 years of life becomes difficult if these initial years were characterized by disorganized, absent, or abusive primary caregivers” [39]. Some of our informants are struggling and the reason might very well be that their caring environment might not have been sufficiently stable, predictable or supportive during their first years. The reason for the inconsistent caregiving environment might besides poverty and overburdened families, affected by AIDS, also be complicated internal family dynamics.

## Implications

As we know that the extended family system is the most important contributor to the survival of these children and adolescents, our efforts to prevent the development of mental disorders on the basis of our findings should go from individual to system directed interventions by strengthening the families, both financially and by education. Reinherz and colleagues [40] have underlined that “…a positive adolescent family milieu is related to both adaptive outcomes and a reduced likelihood of serious difficulties, including mental and behavior problems”, while Kumpfer & Alvarado [41] have emphasized that negative adolescent behavior can be avoided through early intervention by strengthening family relationships and dynamics. By strengthening the family, the child and adolescent will have a better opportunity to develop necessary psychological resilience factors and become less vulnerable. Besides a detected need of screening and treatment for mental disorders in HIV service provision [42] the preventive efforts consequently should target the strengthening of AIDS affected families: «The capacities of families to protect children and to compensate for their loss of caregivers, security, possessions and the like, is highly dependent on the social context, most especially, pervasive and enduring poverty and labour migration [43]. Consequently, an effective response to the challenge is to work for a basic income security as well as access to health and education services for AIDS affected families. This way more stability and predictability could be created, while the health services also must be aware of children and adolescents living in conditions with complex family dynamics and assist in the efforts to create a nurturing environment. Disclosure and acceptance of one’s status also must be targeted, as this often turns out to be a problematic issue. Being difficult for the adolescents to disclose their problems to older strangers like health personnel, it might be worth of constructing peer support groups as suggested by some of our informants and which has been shown to be effective by for example Mupambireyi et al. [9]. This intervention also seems appropriate given the shortness of well-educated health workers. Uganda has currently approximately 35 psychiatrist and even fewer clinical psychologists for a population of approximately 34,5 million people in 2014 [44]. In light of the absence of sufficient qualified clinical psychologists/psychiatrists the strengthening and incorporation of existing social networks like family and peers seems to be inevitable in the efforts to improve the developmental conditions of the children and adolescents.

## Conclusion

It is time to accept that it is not enough that HIV-infected children and adolescents survive physically and have a reasonable health, but that their and their families’ conditions have psychosocial consequences, hampering and preventing the possibility of developing sufficient psychological resilience, which might end in mental disorders and decreased life-quality. Secure attachment and positive self-concept are decisive protective mechanisms when these adolescents face their life challenges. Their existence and strength might be crucial in order to survive both physically and mentally. Therefore, it is not sufficient to diagnose and treat individuals, but one has also to focus on the care environment and trajectory as well as inherent conflictual family dynamics in both treatment and prevention. Awareness on attachment disorders might change the character of the therapeutic and preventive efforts significantly, as it would change the focus towards strengthening the development of psychological resilience. Peer groups could contribute further by both normalizing the situation and thus contribute to the development of a positive self-concept.
